# Evaluation of gene-based association tests for analyzing rare variants using Genetic Analysis Workshop 18 data

**DOI:** 10.1186/1753-6561-8-S1-S9

**Published:** 2014-06-17

**Authors:** Andriy Derkach, Jerry F Lawless, Daniele Merico, Andrew D Paterson, Lei Sun

**Affiliations:** 1 Department of Statistical Sciences, University of Toronto, Toronto, Ontario M5S 3G3, Canada; 2 Department of Statistics and Actuarial Science, University of Waterloo, Waterloo, Ontario N2L 3G1, Canada; 3 Division of Biostatistics, Dalla Lana School of Public Health, University of Toronto, Ontario M5S 3G3, Canada; 4 The Centre for Applied Genomics, The Hospital for Sick Children, Toronto, Ontario M5G 1L7, Canada; 5 Program in Genetics and Genome Biology, The Hospital for Sick Children, Toronto M5G 1X8, Canada

## Abstract

The focus of our work is to evaluate several recently developed pooled association tests for rare variants and assess the impact of different gene annotation methods and binning strategies on the analyses of rare variants under Genetic Analysis Workshop 18 real and simulated data settings. We considered the sample of 103 unrelated individuals with sequence data, genotypes of rare variants from chromosome 3, real phenotype of hypertension status and simulated phenotypes of systolic blood pressure (SBP) and diastolic blood pressure (DBP), and covariates of age, sex, and the interaction between age and sex. In the analysis of real phenotype data, we did not obtain significant results for any binning strategy; however, we observed a slight deviation of the *p*-values from the uniform distribution based on the protein-damaging variant grouping strategy. Evaluation of methods using simulated data showed lack of power even at the conservative level of 0.05 for most of the causal genes on chromosome 3. Nevertheless, analysis of *MAP4 *produced good power for all tests at various levels of the tests for both DBP and SBP. Our results also confirmed that Fisher's method is not only robust but can also improve power over individual pooled linear and quadratic tests and is often better than other robust tests such as SKAT-O.

## Background

Next-generation sequencing (NGS) technology provides rich data for the analysis of the role of rare variants in complex human diseases and traits. Because of the low power associated with analyzing one single rare variant, many pooled association tests have been proposed for joint analysis of a group of rare variants. The methods proposed so far include the earlier linear statistics that are powerful when most of the variants are causal and have the same direction of effect [1,2], the quadratic statistics that are not sensitive to the direction of effect [3-5], and the more recent hybrid statistics that combine the evidence from the complementary linear and quadratic statistics [6,7]. However, few studies evaluate the different classes of tests, and fewer investigate the impact of different variant grouping strategies on the pooled association analysis, which is the goal of our study here. Using the Genetic Analysis Workshop 18 (GAW18) real and simulated data, we evaluate two commonly used statistics from each of the linear and quadratic classes of tests plus three recently proposed robust statistics, and we investigate five gene-based variant grouping strategies, of which three focus on coding variants.

## Methods

### Association tests for rare variants

We considered 7 association tests recently developed for analyzing rare variants using a sample of unrelated individuals. All methods considered can be described through a vector of statistics, ***S ***= (*S_1_, ..., S_J_*), where

$$S_{i}=\overset{n}{\underset{i=1}{\sum }}\left(Y_{i}-{\hat{Y}}_{H_{0},i}\right)G_{ij}$$

and *J *is the total number of single-nucleotide polymorphisms (SNPs) in the bin or group of variants under study, *Y_i _*is the phenotype value of the *i*th individual*, i = 1,...,n, G_ij _*is the number of rare alleles for the *i*th individual at the *j*th SNP, and ${\hat{Y}}_{H_{0},i}$ is the fitted phenotype value of the *i*th individual under the null hypothesis of no association between the phenotype *Y *and genotypes *G *of the group of variants under study. The fitted phenotype values can depend on a set of covariates *X*.

The 7 "pooled" association test statistics are functions of the vector ***S***, aggregating information across the *J *variants. They include *two linear statistics *of the form

$$W_{Lw}=\overset{J}{\underset{j=1}{\sum }}w_{j}S_{j},$$

where *W_L1 _*uses equal weights [1] and *W_Lp _*uses minor allele frequency- (MAF, *p*) based weights [2], *two quadratic statistics *of the form

$$W_{Q}=S^{\prime }AS,$$

where the C-alpha statistic, $W_{C}=\overset{J}{\underset{j=1}{\sum }}S_{j}^{2}$, uses *A = I *[3], and the Hotelling statistic, *W_H_*, uses the inverse of the estimated covariance matrix of ***S ***under the null hypothesis [4,5], and *three robust hybrid statistics*, SKAT-O [6], minimum-p and Fisher's statistics [7], which combine the association evidence from complementary linear and quadratic statistics. The minimum-p statistic is

$$W_{\text{min}}=\text{min}\left(p_{L1},p_{C}\right),$$

and Fisher's statistic is

$$W_{F}=-\text{log}\left(p_{L1}\right)-\text{log}\left(p_{C}\right),$$

where *p_L1 _*and *p_C _*are *p*-values, respectively, of the linear *W_L1 _*and quadratic *W_C _*tests. The SKAT-O statistic is similar in nature to the minimum-p statistic [6,7].

### Gene annotation and data analysis

For the purpose of this study, we focused on the 103 unrelated individuals with NGS data from the GAW18, rare variants from chromosome 3, the first of four measurements for systolic blood pressure (SBP), diastolic blood pressure (DBP) and hypertension status, and covariates age, sex, and the interaction between age and sex. Rare variants were defined as SNPs with MAF of 0.05 or less, estimated from the 103 unrelated sequenced individuals. The analysis of the real data focused only on the hypertension status; the analysis of the simulated response data studied premedicated SBP and DBP values (for medicated individuals we increased their values of SBP and DBP by 6.2 and 7.9, respectively, as specified in the provided answers) as well as hypertension status. Last, we analyzed the continuous and dichotomized values of simulated phenotype Q1, which is unrelated to the genotypes, to evaluate the type I error of the 7 statistics.

The selection of groups of rare variants for pooled association analyses is critical, in terms of statistical power, for all methods [1-7]. We used several software packages to annotate the sequenced SNPs. Gene mapping and variant type annotations were done with ANNOVAR [8]. Variant impact predictions were generated using SIFT [9] and PolyPhen [10], combined scores were based on CONDEL [11], and conservation scores were downloaded from UCSC (placental-mammal) [12]. More details of the annotation procedures are presented in another GAW18 paper by Nalpathamkalam *et al *[13].

Based on the variant annotation, we considered a gene-based approach using three strategies to group/bin the variants within each gene. We grouped variants that belong to the same gene and are of the same annotation type, that is, (a) coding variants, (b) protein-changing variants, and (c) protein-damaging variants. We note that (c) is a subgroup of (b), and (b) is a subgroup of (a).

We obtained *p*-values for each of the 7 statistics by parametric bootstrap [4,5]. For the continuous response, we first fitted a linear regression model that includes age, sex, and interaction between age and sex, which corresponds to the null hypothesis that the genotypes of *J *SNPs are not associated with the continuous response variable (SBP or DBP) given the other covariates. Similarly, we fitted logistic regression models with the same set of covariates when hypertension status is considered as the response. We then generated bootstrap samples from the fitted models and calculated the 7 test statistics for each sample. Finally, we obtained the empirical *p*-value for each test as the proportion of bootstrap samples with statistics more extreme than the one calculated from the original data. For each statistic and a group of SNPs, we initially used 1000 bootstrap samples to estimate the *p*-value. If the *p*-value was less than 0.01, we used an additional 100,000 bootstrap samples to estimate the *p*-value more accurately. For the *p*-value of SKAT-O, we used the available SKAT-O package [6].

## Results

### Gene annotation

Based on the sequence data on chromosome 3, there were 7435 high-quality variants annotated as coding variants, 4099 as protein-changing variants, and 1791 as protein-damaging variants (Table 1). However, these numbers were reduced with the restriction of MAF of 0.05 or less (Table 1).

**Table 1:** Descriptive statistics of different grouping or binning strategies based on annotations of sequence variants on chromosome 3

Strategies of grouping variants in a gene	Total # of variants	Restricting to variants with MAF ≤ 0.05			
		
		# of genes with ≥1 variant	# of genes with ≥2 variants	Average # of variants per gene with ≥1 variant	Average MAF
**a. Coding variants**	7435	900	690	4.34	0.012

**b. Protein changing**	4099	729	479	2.95	0.011

**c. Protein damaging**	1791	462	210	1.94	0.011

**d. Protein changing or conservative T1**	15,326 (4099 + 11227)	1034	841	8.17	0.011

**e. Protein damaging or conservative T2**	5987 (1791 + 4196)	735	438	4.31	0.012

Descriptive statistics for rare variants with minor allele frequencies of 0.05 or less were constructed from the sample of 103 unrelated individuals. The number of genes and average number of variants per gene were slightly reduced when we analyzed the real data because the number of individuals was reduced to 96 after consideration of missing phenotype and covariates.

### Real data

We evaluated the type I error for all 7 methods using Q1 as the outcome. Because the *p*-values for all methods except SKAT-O were based on parametric bootstrap resampling, we observed correct type I error for all the 6 methods as expected. We also observed correct type I error for SKAT-O (results not shown).

**Figure 1:**
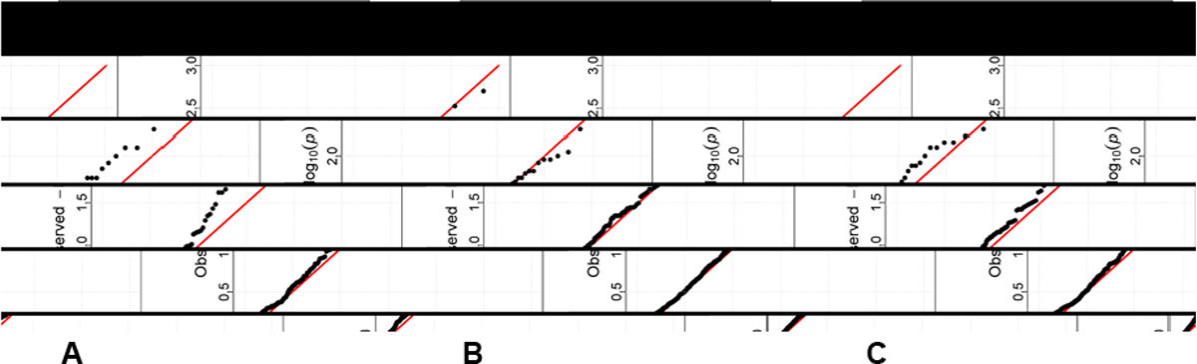
**Quantile-quantile plots of -log10 (*p*-values) based on Fisher's statistic for all genes with 2 or more rare variants (reduced) in real data**. (**A**) Coding (reduced). (**B**) Protein change (reduced). (**C**) Protein damage (reduced). See Table 1 for detailed variant annotation and binning strategies **A **to **C**. The *p*-values were obtained using the parametric bootstrap method as described in the text for hypertension status.

In the analysis of hypertension status based on the real data, none of the genes appeared to be statistically significant using a crude Bonferroni correction for multiple testing (0.05/888 = 5.63 × 10^−5 ^for coding, 0.05/720 = 6.94 × 10^−5 ^for protein changing, and 0.05/460 = 1.1 × 10^−4 ^for protein damaging). However, we observed that with a more refined selection of SNPs, such as the protein-damaging variants compared with the coding variants, there are deviations (i.e., more small *p *values) from the expected Unif (0,1) distribution for *p*-values of all 7 tests, suggesting association. As an example, Figure 1 presents the quantile-quantile plots for Fisher's statistic for the set of genes with at least 2 variants (denoted as *reduced *compared with the number of genes with at least one variant) based on each of the three binning strategies (a) to (c) in Table 1.

### Simulated data

The last stage of our analysis focused on the simulated data to assess the performance of the 7 methods in terms of power. As a proof of principle, the results presentation here focuses on binning strategy (a). Of the 31 causal genes on chromosome 3 influencing SBP or DBP, 25 genes had at least one rare variant annotated as coding and polymorphic in the sample of 103 unrelated individuals. For most of these causal genes, none of the 7 tests had reasonable power (10% ore more) for any of the three phenotypes even at the conservative type 1 error level of 0.05 (Table 2).

**Table 2:** Empirical power for the 7 association tests using simulated phenotype data

Gene	Total # of rare variants	# of causal rare variants	Methods						
			**Linear W_L1_**	**Linear W_Lp_**	**Quadratic W_C_**	**Quadratic W_H_**	**Minimum W_min_**	**Fisher's W_F_**	**SKAT-O W_SKAT-O_**

***Outcome = SBP***									

*BTD*	6	2	0.29	**0.33**	0.15	0.15	0.24	0.27	0.25

*MAP4*	8	4	0.96	0.93	0.96	0.91	0.97	**0.99**	0.96

*ABTB1*	6	0^1^	0.05	0.05	**0.16**	0.09	0.12	0.12	0.08

*PPP2R3A*	8	2	0.25	0.18	0.21	0.13	0.28	**0.32**	0.19

*GPR160*	1	0^1^	0.19	0.19	0.19	0.19	0.19	0.19	0.19

***Outcome = DBP***									

*BTD*	5	2	**0.36**	0.35	0.16	0.11	0.29	0.31	0.26

*MAP4*	8	4	0.90	0.88	0.86	0.73	0.90	**0.94**	0.91

*ABTB1*	6	0^1^	0.10	0.06	**0.14**	0.05	0.11	0.11	0.08

*PPP2R3A*	8	2	0.06	0.04	**0.20**	0.11	0.14	0.15	0.09

*GPR160*	1	0^1^	0.27	0.27	027	0.27	0.27	0.27	0.27

***Outcome = hypertension status***									

*PDCD6IP*	6	0^1^	0.17	0.14	0.17	0.13	0.19	**0.20**	0.18

*FLNB*	13	4	0.07	0.07	0.10	**0.11**	0.08	**0.11**	0.08

*SENP5*	4	1	0.06	0.05	0.07	**0.16**	0.08	0.07	0.06

Two continuous phenotypes, systolic blood pressure (SBP) and diastolic blood pressure (DBP), and one binary phenotype, hypertension status, were analyzed. Rare variants (minor allele frequency ≤0.05) were grouped by gene and annotated as coding (strategy a in Table 1). All causal variants have the same direction of effect by the Genetic Analysis Workshop 18 simulation design. Level of tests was set to 0.05 because of a lack of power at a more stringent level. Genes presented are the ones with maximum power (bolded) among the 7 tests greater than 10% at the 0.05 level.

^1^"Power" for these genes with no causal variants are attributable to linkage disequilibrium between non-causal rare variants in these genes and causal variants in other genes (see text for details).

We first note that *ABTB1, GPR160*, and *PDCD6IP *do not have causal rare variants, but the estimated power for some tests is larger than 10%. Such results are due to linkage disequilibrium (LD) between non-causal rare variants in genes with causal rare variants from other genes. For example, the non-causal rare variant with coordinate 127395914 in *ABTB1 *is highly correlated with the causal variant with coordinate 48040284 in *MAP4*. Similarly, we observed that the genotype of a single non-causal rare variant (169801953) in *GPR160 *is identical to the genotype of a causal rare variant (47913455) in *MAP4 *in the sample of 103 individuals considered.

Results in Table 2 clearly show that individual pooled linear and quadratic statistics can have substantial difference in power even when all causal rare variants have effects in the same direction. For example, for the causal *BTD *gene, the estimated power for the two linear statistics was significantly larger than those for the two quadratic statistics (for both SBP and DBP), but the pattern is reversed for *ABTB1 *(for both SBP and DBP) and *PPP2R3A *(for DBP). In contrast, the three hybrid statistics, *W_min_, W_F_, *and *W_SKAT-O_*, are robust: for each gene, they are comparable in terms of power to the method with the maximum power for that gene. We also observed that Fisher's method, which combines *p*-values from the linear and quadratic test statistics, has better power in most cases than the other two robust statistics, minimum-p and SKAT-O, and it is the best or the second best option in many cases.

**Figure 2:**
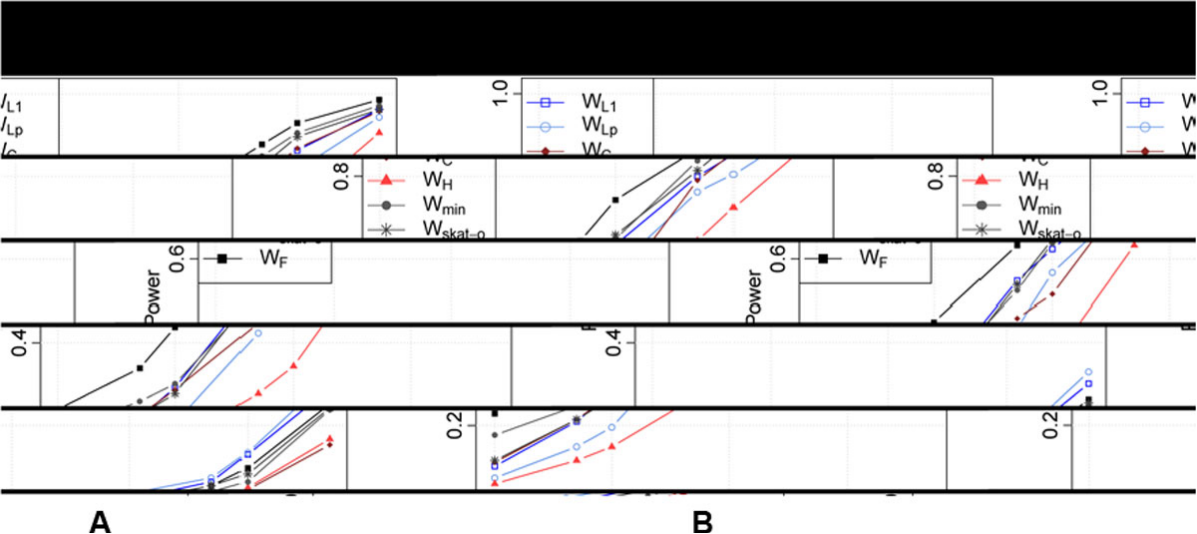
**Receiver operating characteristic curve examining the relationship between power and type I error for the 7 tests**. (**A**) ***BTD***.(**B**) ***MAP4*. **The phenotype is systolic blood pressure (SBP). For the *MAP4 *gene, 4 of the 8 rare variants have a causal effect on SBP; in total, they explain 5.8% of variation in SBP.

Results in Table 2 also show that, with the exception of *MAP4*, all tests have low power even at the 0.05 level; adjusting for multiple testing will further decrease power. Focusing on *MAP4 *and *BTD*, the two genes with the largest power for SBP, we investigated the relationship between power and level of the tests using a receiver operating characteristic (ROC) curve (Figure 2). For *MAP4*, the Fisher's method consistently outperforms all other tests, and power is above 20% even at the 10^−5 ^level; for *BTD*, all tests have power less than 20% even at the 0.01 level. We observed a similar pattern with DBP; analyses for hypertension were not considered because of the low power even at the 0.05 level.

## Discussion

We investigated several gene-based grouping strategies for rare variants and analyzed both the real and simulated phenotype data. We observed that further restriction of rare variants based on annotation is promising (e.g., from coding to protein damaging); however, we did not observe statistically significant results after adjusting for multiple hypothesis testing. The strategies presented so far focused on coding variants. We also considered two other strategies that include non-coding variants but restricted to variants that belong to conservation tier 1 group (T1) (PhastCons score >0 and PhyloP score >1) and tier 2 group (T2) (PhastCons >400 and PhyloP score >1.5) (see Nalpathamkalam *et al *[13] for more details on these annotation strategies). These lead to grouping variants from the same gene, which are (d) protein-changing or conservative T1 variants with 11,227 high-quality variants and (e) protein-damaging or conservative T2 variants with 4196 high-quality variants (Table 2). These additional strategies could be more powerful than the coding-only strategies because causal rare variants from regulatory regions could be added to the analysis. However, we note that the simulated causal variants in GAW18 were all in or near gene. When strategies (d) and (e) were considered in our additional analysis with the real binary hypertension phenotype, we did not observe significant improvement in terms of departure of the empirical *p*-value distribution from the null distribution. We did, however, observe that top-ranked genes differ considerably among the different binning strategies, further confirming the practical importance of annotation in analyzing a group of rare variants [5].

In the analysis of simulated data, as a proof of principle, we focused on the comparison of various methods using binning strategy (a). Most of the genes with causal variants had poor or no power even at the conservative level of 0.05. This is due to the small sample size, small effect sizes, and perhaps our binning strategy. Nevertheless, analysis of *MAP4 *produced good power for all tests at various levels of the tests for both DBP and SBP. We also noticed that studies with hypertension status as a response variable generally had lower power than studies with SBP and DBP, indicating that dichotomization of blood pressure into just two groups masks the effect of the genes. Our results also confirmed that Fisher's statistic is not only robust but can also improve power over individual pooled linear and quadratic tests and is often better than SKAT-O, which relies on the minimum *p*-value principle [7]. The results here are consistent with what has been reported in the literature (e.g., [4,5]): power differences between linear and quadratic statistics can be substantial, and robust statistics are needed to provide consistently acceptable power across different genetic settings. In addition, Fisher's statistic performs best when, as here, "the majority of the causal variants have the same direction of effect (either deleterious or protective)" [7]. Finally, we show that LD between causal variants in one gene and non-causal variants in nearby genes can result in potential confounding and apparent false positives.

## Competing interests

The authors declare that they have no competing interests.

## Authors' contributions

AD, JFL, and LS created the overall study design. AD did the overall analysis. DM and ADP created the gene annotation design and did the analysis. AD, JFL, and LS drafted the manuscript. All authors read and approved the final manuscript.
